# Drug preparation, injection, and sharing practices in Tajikistan: a qualitative study in Kulob and Khorog

**DOI:** 10.1186/s13011-016-0065-2

**Published:** 2016-06-02

**Authors:** David Otiashvili, Alisher Latypov, Irma Kirtadze, Umedjon Ibragimov, William Zule

**Affiliations:** Addiction Research Center, Alternative Georgia, 14a Nutsubidze Str., Office 2, 0177 Tbilisi, Georgia; Global Health Research Center of Central Asia, Columbia University, New York, USA; The Central Asia Program, Institute for European, Russian, and Eurasian Studies, The Eliott School of International Affairs, George Washington University, Washington, DC USA; Business School, Ilia State University, Tbilisi, Georgia; Rollins School of Public Health, Emory University, Atlanta, GA USA; RTI International, Research Triangle Park, NC USA

**Keywords:** Injection drug use, Indirect sharing, Infection risks, Tajikistan

## Abstract

**Background:**

Sharing injection equipment remains an important rout of transmission of HIV and HCV infections in the region of Eastern Europe and Central Asia. Tajikistan is one of the most affected countries with high rates of injection drug use and related epidemics.The aim of this qualitative study was to describe drug use practices and related behaviors in two Tajik cities – Kulob and Khorog.

**Methods:**

Twelve focus group discussions (6 per city) with 100 people who inject drugs recruited through needle and syringe program (NSP) outreach in May 2014. Topics covered included specific drugs injected, drug prices and purity, access to sterile equipment, safe injection practices and types of syringes and needles used. Qualitative thematic analysis was performed using NVivo 10 software.

**Results:**

All participants were male and ranged in age from 20 to 78 years. Thematic analysis showed that cheap Afghan heroin, often adulterated by dealers with other admixtures, was the only drug injected. Drug injectors often added Dimedrol (Diphenhydramine) to increase the potency of “low quality” heroin. NSPs were a major source of sterile equipment. Very few participants report direct sharing of needles and syringes. Conversely, many participants reported preparing drugs jointly and sharing injection paraphernalia. Using drugs in an outdoor setting and experiencing withdrawal were major contributors to sharing equipment, using non-sterile water, not boiling and not filtering the drug solution.

**Conclusion:**

Qualitative research can provide insights into risk behaviors that may be missed in quantitative studies. These finding have important implications for planning risk reduction interventions in Tajikistan. Prevention should specifically focus on indirect sharing practices.

## Background

Injection drug use and related infections represent major public health problems in Tajikistan and other Central Asian republics [1, 2]. In Tajikistan, there are an estimated 25,000 (range 20,000-30,000) people who inject drugs (PWID) [3]. Unsafe injecting practices among PWID account for about half of all HIV infections in Tajikistan [4]. The estimated prevalence of HIV among PWID is 13 % ranging between 0.7 to 27 % depending on a locality [5, 6]. Countrywide hepatitis C virus (HCV) prevalence among PWID is 23 % with some locations reaching 47 %. With the population of 8 million people and per capita gross national income (GNI) of 1060 USD Tajikistan is classified as a lower-middle income country by the World Bank [7] and relies heavily on international funding for its response to HIV/AIDS related problems. As of 2014, 51 harm reduction sites operated throughout the country providing, according to national authorities, HIV prevention services (needle and syringe distribution, condom distribution, and voluntary counseling and testing) to 11,993 PWID [5]. In 2014, these harm reduction sites distributed 4,944,987 needles and syringes. Tajikistan also has 6 opioid substitution programs (two in Dushanbe, and one each in Kulob, Khudjand, Khorog and Kurgan-Tube) covering 577 patients with opioid dependence [5]. The Global Fund to Fight AIDS, Tuberculosis and Malaria (GFATM) funds all these HIV prevention programs for PWID.

Sharing needles, syringes and other drug preparation equipment is an important route of transmission of both HIV and HCV infection. Increases in prevention program coverage in the region have improved knowledge and reduced risk behaviors [4, 8], however, these changed have rarely resulted in reductions in new HIV and HCV infections among drug PWID. To the contrary, in many countries in Eastern Europe and Central Asia HIV incidence has been rising [9], and research focusing on in-depth understanding of behavioral risks associated with drug injecting in Tajikistan and other Central Asian countries remains scant. This qualitative study aims to fill this gap by investigating drug preparation, consumption and sharing routines to understand risk factors and previously unexplored aspects of transmission risk that may contribute to the high rates of HIV and HCV among PWID in Tajikistan.

## Methods

### Sampling and recruitment

In May 2014 we worked with local needle and syringe programs (NSPs) in Khorog and Kulob, Tajikistan to recruit 100 PWID for focus group discussions. We used a purposive and convenience sampling approach to recruit participants. Study eligibility criteria included: a minimum age of 18 years, injecting drug use in the past month and ability to speak Tajik or Russian languages.

### Ethics approval

The study received ethical approval from the Republican Committee of Medical Ethics of Tajikistan and RTI International’s Office of Human Research Protection (Federalwide Assurance No. 3331, IRB ID No. 13180). All participants provided oral informed consent, and received the equivalent of 5 US dollars for their participation.

### Interviewing

Focus group discussions were held at the NSP site in each city. Two experienced researchers facilitated each focus group, which lasted from 60 to 90 min. The research team developed the focus group discussion guide based on team members’ knowledge of the topic and understanding of the local drug scenes. Topics covered during the focus groups included detailed descriptions of the drug preparation and consumption practices among PWID in each city.

### Qualitative analysis

All discussions were audio recorded, transcribed verbatim in Russian or Tajik, translated into English and catalogued in NVivo v.10 software. A research staff member, fluent in Tajik, Russian and English, transcribed and translated all of the recordings; the two researchers who facilitated the focus groups discussions compared the English versions with the originals to ensure the accuracy of the translations. The research team used a coding frame derived from the focus group guide to code the transcripts, which were then analyzed thematically for drug preparation and consumption behaviors and factors influencing these behaviors. Coding and analysis were done independently by two researchers in parallel and were later discussed and agreed upon by all research team members.

## Results

We conducted 12 focus group discussions (6 per city) with six to 10 participants per group and 100 total in Khorog (*n* = 47) and Kulob (*n* = 53). All 100 participants were male and ranged in age from 20 to 78 years with a median age of 43 years. The mean number of injections per week was 15 (range 1–35); 75 % reported obtaining needles and syringes from NSPs.

Below we present the results of the analysis in line with the key themes identified - availability and purity of drugs, preparation and injection practices, and injection-related risks.

### Drugs consumed, availability, price and purity

In both cities, the vast majority of participants reporting injecting cheap Afghan heroin almost exclusively, however, a few participants mentioned injecting acetylated opium (“*khanka*”). According to focus group participants, a very small fraction of heroin users, mostly beginners, use heroin by smoking or snorting instead of injecting it. Most of those who smoked or snorted heroin when they started reported shifting to injection because of the limited supply of heroin and constantly increased tolerance towards drug. A few participants in Kulob mentioned a specific type of drug (“crystal” or “synthetic” heroin, “Chinese” heroin), which is sold as heroin but looks slightly different from the regular heroin and *“glitters like a glass”*. Solutions of this type of “heroin” *“become like alabaster in the syringe”*, can cause sharp pain in a limb, and can increase the probability of overdose. The chemical composition of this “crystal heroin” is unknown. One participant mentioned injection of Tropicamide eye drops to “*relieve agonies (withdrawal syndrome)”*.

Participants in all focus-group discussions mentioned limited availability (compared to previous years) of heroin on a local black market. Almost all participants reported that suppliers heavily adulterate heroin available for end users. Most participants believe that drug dealers in Aghanistan and Tajikistan adulterate heroin, with dealers in Tajikistan being blamed the most. According to participants, dealers mix heroin with sugar, medicines such as antihistamines, specifically Dimedrol (Dyphenhydramine), non-steroid painkillers (Acetaminophen, Analgine, Baralgine), psychotropic drugs (Zopiclone), calcium chloride and oral rehydration salts. Some participants reported that drug dealers may use such admixtures as flour, lime powder, alabaster. When heroin is in short supply, drug dealers cut it more heavily. The retail price of heroin depended on a purity and quantity purchased, but on average was about $10 (50 Somoni) per gram, while one average dose (“shponka”, approximately 0.15 g) was about $2 (10 Somoni) at the time of data collection. More pure heroin sold for $20 (100 Somoni) per gram.

### Preparation of drug solution

Many participants reported that they usually injected in groups of two or three people, usually the same people and rarely with strangers. They reported preparing heroin in a variety of metal (spoons, metal cups from safe injection kits), glass (bottles, vials) and plastic (bottle caps) mixing vessels. Many participants mentioned mixing heroin with other medicines. *“Half [of people] use Dimedrol, half do not”* (FGD#8, Khorog), usually adding one or two crushed tablets to the cooker per dose of heroin. The main reason for adding Dimedrol is to potentiate the effects of heroin *“so the high is stronger”* (FGD#11, Khorog), particularly if the amount of heroin is small or it is heavily adulterated. However, some participants reported adding Dimedrol to avoid nausea caused by heroin. Although people usually used Dimedrol tablets, they also use liquid Dimedrol in ampules, which they substitute for water to dissolve the heroin. Although somewhat less common, people occasionally add non-opioid painkillers containing Metamizole (Analgin) or Paracetamole. Some participants reported using alcohol to potentiate the effect of heroin and “*get more high (from a dose)”*.

Many participants reported injecting with sterile water in ampules provided by NSPs. When sterile water is not available or a person is in withdrawal, people may use water from a tap, river, ditch or a rainwater puddle. People usually use from one-half ml to one ml of water per dose of heroin, using more water when two or more people inject together. Some participants believe that adding more water to the solution makes it less concentrated resulting in weaker effect, while others preferred to add more water making it appear like a larger dose, which is more comforting psychologically.

Sometimes, users filter and heat the drug solution but these steps can be skipped when the powder is easily soluble. The drug solution must be heated when *“…there are kinds of drugs that do not melt, do not get dissolved”* (FGD#8, Khorog) and to make sure “*there is no infection there, so all extra stuff gets burned there”* (FGD# 10, Khorog)*.* Some participants reported that boiling causes the drug solution to thicken and gel if it contains certain impurities, making it impossible to inject. A few participants also believed that injecting hot, uncooled drug solution can cause hepatitis and/or other liver damage. The overall agreement was that *“…heroin is a universal drug; if you want you can boil the solution, or mix it with cold water, or with water from a ditch. It will go”* (FGD#3, Kulob).

Participants reported using filters supplied by the NSP or using improvised filters from a cotton swab or a cigarette filter. Prior to drawing drug solution into a syringe through a filter, many participants reported removing the needle to reduce the risk of the needle becoming clogged or accidentally jamming it into the cooker and dulling it. Others draw the solution through the needle using a piece of cotton rolled over the tip of a needle as a filter. Filtering the drug solution reduces the presence of solid particles that can clog the needles, which allow people to inject with thinner needles that do less damage to veins.

### Drug division and injection

The rules regarding division of heroin purchased by two or more people vary depending upon situational factors. Often, the person who contribute the most money receives the largest share of heroin. In other cases, people apportion the heroin based on individual dosage needs (e.g., tolerance, presence of withdrawal symptoms, etc.). Despite these “rules” people often divide the drug equally regardless of who paid how much - for example, between good friends, or if a person paid less (or no money) but helped to find and to buy heroin. Cheating during division of drug is not common and is not tolerated. The rules of drug division are negotiated in advance. Although heroin can be divided either before dissolving (the powder is divided) or after dissolving (the liquid is divided), none of the focus group participants reported dividing powder heroin. When liquid is divided, PWID may prepare the drug in a common container and then take turns drawing it into their syringes. Alternatively, they may draw everything into one relatively large volume syringe and then transfer it into individual syringes by frontloading (i.e., inserting the needle of one syringe into the tip of another syringe (with the needle detached from the latter syringe). Frontloading may also happen if one of partners accidentally draws more solution than agreed.

Many participants mentioned that they check if the needle is in the vein by drawing some blood into the syringe, calling this practice *“kontrol’ka”* in Russian slang language. If the needle hub is translucent, they need to draw less blood; if it is opaque, they need to draw more blood into the syringe. Many participants reported the practice of “flushing” any residual drug solution from the syringe by drawing additional blood into it and reinjecting the blood. Others may add some more water to the syringe to use the leftover. However, a substantial share of participants, primarily in Khorog, stressed that they do not try to use the drug leftovers because it does not matter much for them.

### Dosing and frequency

For many heroin users, the amount of money they have strongly determines the number of times that they inject heroin and the amount of heroin in each injection. Some participants mentioned that PWID would inject as much heroin as they can get, up to 3–5 g in a day. Others mentioned that because heroin is heavily adulterated, people needle inject larger amounts of it. When heroin users have trouble obtaining heroin, they often use less, perhaps only a fraction of a gram per day. In general, the most common frequency of injection is 2–3 times per day. Participants reported that as with the daily dosage, the frequency of injection depends on the amount of heroin available. When little heroin can be found, people only inject 1–2 times a day. Many participants said that when plenty of heroin is available, most people will consume it all in one or two days by injecting more frequently, 5–6 times a day or every 2–3 h. Some participants indicated that they have to inject more frequently due to the lower potency of heroin.

### Use of sterile equipment

Focus group participants agreed that NSPs are the primary source of needles and syringes for most PWID, but many PWID also obtain needles and syringes from pharmacies. The choice of source depends on several factors: location and distance, time (NSP do not work on weekends or after regular office hours), range and quality of the injecting equipment, and availability of money to buy syringes. In some cases PWID get syringes from a relative or a neighbour who works in a hospital. The main advantages of NSP are free injecting equipment and the possibility of getting many syringes (although in Kulob some participants mentioned limited daily quotas of three syringes) as well as the convenience of receiving syringes from outreach workers (without the need to visit the NSP site). It was acknowledged that unlike other health care providers, NSP staff have nonjudgmental and empathetic attitude towards PWID. However, NSP disadvantages are limited hours and days of operation and the need to commute to the site if outreach workers are not around.

Accordingly, many participants reported that they also buy syringes in pharmacies. This is particularly common if they are experiencing withdrawals from heroin and are unwilling to wait for an outreach worker or travel to the NSP site. Unlike NSP, pharmacies are open for extended hours seven days a week, are located everywhere and can be easily accessed when people need syringes in a hurry. In addition, syringes are relatively cheap (0.25 Somoni, or approximately 0.05 USD as per exchange rate in 2014) and most pharmacists will sell them. Nevertheless, several participants reported that some pharmacists scold PWID who try to buy syringes, and some pharmacists refuse to sell syringes. Pharmacists may suspect that a buyer is a drug injector if the latter asks for both a syringe and Dimedrol. Another concern with the pharmacies is harassment by police who may wait outside of pharmacies looking for potential PWID. Some pharmacists may report suspected PWID to police. The quality of syringes in pharmacies may also be lower as compared to NSP since pharmacists do not take into account specific needs of PWID while ordering syringes.

### Unsafe injection practices

Almost all participants in Khorog and many in Kulob reported using sterile needles and syringes only. PWID appear to have accurate and sufficient knowledge about risks of sharing needles and syringes. Many reported that they had shared needles and syringes before they were aware of HIV transmission risks and before sterile instruments were widely available through NSPs. As stated by one participant *“…since [NSP] started working, we use one thing only once, then throw it away*” (FGD#1, Kulob). Participants reported that PWID rarely share syringes anymore and generally only in situations when someone is in acute withdrawal and sterile syringes are not available.

*“…it happens, say, they are sitting there, and no syringes, and he is in agony [from withdrawal], so he would take [the used syringe] from anywhere, he is sick [from withdrawal], he has no money even to buy a syringe, or to go [to get a syringe], he is in agony…”* (FGD#8, Khorog).

In some cases, one *“…would shoot half of it and then would give it to another one, so he shoots up another half [of the solution]”* (FGD#12, Khorog). In other situations *“…if there is no [other syringe], one would hit, then another one would wash it and draw [the rest of solution] for himself”* (FGD#1, Kulob). Some participants mentioned replacing the needle and injecting with a shared syringe.

*“We would clean it, then replace the needle, that’s it. (Many voices): just the needle, just would replace the needle, that’s it”* (FGD#11, Khorog).

A number of participants mentioned that many young injectors lack awareness and knowledge regarding injection risk behaviors. Consequently, young injectors may share syringes and engage in indirect sharing practices.

*“…these young ones, beginners, I personally saw them [sharing]. …he also was there, sitting, and he was without a syringe, so he is like, “Bro, give me one, so I shoot too”. And I told him “What if I am sick, if I’ve got that disease?” But he, he is young, so he is like, “C’mon, c’mon, it won’t be a problem”* (FGD#10, Khorog).

Although PWID rarely engage in direct syringe sharing, sharing other injection equipment (e.g., mixing vessels [i.e., cookers], cotton filters, rinse water) occurs much more frequently. Several participants report that many PWID use a shared container to prepare heroin. However, participants did not perceive any transmission risks because each person uses his own sterile syringe. If a PWID does not have any heroin, he may inject the residual heroin that remains trapped in a cotton filter from a previous injection, calling it “*vtoryak*” (second-hand drug) in Russian slang language. This may introduce risk if they use someone else’s *vtoryak*. In particular, cottons may be used by “*… a beginner, when he starts [to use drugs]…and his dose is small, …so vtoryak is enough for him…”* (FGD#10, Khorog). Moreover, a number of respondents reported on injecting equipment being re-used. According to the participants, after the injection they may rinse their own used syringes and hide them in some places to use later when no clean syringes are available. PWID perceive re-injection with one’s own syringe as acceptable and safe.

*“If it is a situation where there is no [syringe], when you don’t have one, then I, for example, hide some [syringes] at the backyard of my house, so I know they are there, I would go take them, rinse them. They would be there for 10 days, 20 days. However, I will rinse them, they are mine, so I’ll shoot with them. But I would never use with someone else’s [syringe]”* (FGD# 6, Kulob).

When syringes are reused, repeated rinsing with water is a common practice. Some participants reported using alcohol swabs provided by NSP for superficial cleaning of syringes when no water is available. While reusing one’s own does not place a PWID at risk of HIV or HCV transmission, if a PWID uses the syringe to add water to prepare drugs that will be split with another PWID it may place the other PWID at risk.

The setting in which PWID inject plays an important role in the process of drug preparation and injection. Many PWID prefer to inject in their homes due to perceived safety, and, in most instances, access to new injection equipment or their own equipment as well as sterile or boiled water. However, due to withdrawal symptoms or fear of arrest, PWID may decide to prepare the drug solution and inject on the street or in a secluded location (e.g., riverbank, mountain). In these cases, users often hurry and skip boiling water and filtering the drug solution, and use tap water or water from a ditch to prepare their drugs. Some participants mentioned that PWID in withdrawal prepare the solution right in the syringe, by pouring the drug into the syringe barrel, adding water and shaking it to dissolve the drug mixture.

## Discussion

We did not identify any important differences in drug injection practices and risk behaviors between the two cities. Our findings provide additional support for findings from previous studies, which suggest that access to sterile injection equipment and a safe place to inject are key to reducing injection risk behaviors [10]. It appears that most PWID understand the risks of direct sharing, and direct needle and syringe sharing has become fairly rare. In contrast, the risks associated with indirect sharing through use of common drug injection paraphernalia are poorly understood, and Tajik PWID continue to engage in these behaviors. This is consistent with findings from other studies that found sharing drug preparation equipment was more common than sharing syringes [11–13]. In many drug injecting populations, recognition of HIV transmission risks and scaling up prevention interventions have led to sharp declines in syringe sharing but not in sharing drug preparation equipment. In many locations, users continue to engage in drug preparation and apportioning processes including use of shared cookers, containers, water, syringes for division of solution, front- and backloading, that may introduce contamination with HIV or HCV [13–15]. Utilization and sharing of residual drug that remains in cotton (*vtoryak* in our case) filters after use has been also reported in other settings and has been described as “doing a wash” or “beating the cotton” [12, 14, 16].

These findings have direct practical implications for HIV and HCV transmission prevention. Our findings provide support for current recommendations regarding access to sterile injection equipment and safer injection education programs. However, apart from broadly defining safe injection principles, the focus of such education should be on specific risks associated with different stages of drug preparation and division, and with use of injection paraphernalia. This might be particularly important for HCV prevention since evidence suggests that HCV transmission risk associated with paraphernalia sharing can be comparable to the risk associated with sharing needles and syringes [11, 17]. Several studies have linked HCV transmission to indirect sharing practices in addition to direct sharing of syringes [11, 17–19]. The risk of transmission of HIV through sharing injection paraphernalia seems to be lower compared with the risk of HCV transmission [20]. However, due to the higher prevalence of, indirect sharing practices in many locations, these practices may be of equal or greater importance than direct needle and syringe sharing for both HIV and HCV transmission among PWID.

Our findings suggest that injecting in an outdoor setting and injecting while in withdrawal both contribute to unsafe injecting practices. In these situations PWID, who inject safely in most situations, are more likely to engage in risky practices such as borrowing syringes, sharing preparation equipment, using non-sterile water, and not boiling the drug solution. The presence of withdrawal symptoms and injecting on the street or in a public place have both been reported with unsafe injecting practices in previous studies [13, 21]. In other countries, situational factors contributing to sharing also included perception of safety associated with particular sharing partners, and fear of police and reluctance to carry syringes [13, 15, 22, 23]. In contrast to other regional research we found no elements in drug sharing which would act as a ritual to make friendships closer or strengthen bonds between users [15]. We also found no references to places where many drug users would gather to buy and inject heroin (*yama*, the Russian word for a pit – an analogue of a shooting gallery), which may increase risks for the spread of HIV among PWID. Our research suggests that the group nature of drug acquisition, preparation and injection in Tajik settings is not a result of these activities being perceived by many drug users as a social activity, but rather is a pragmatic response to the illegality of drug use and is often driven by economic and logistical rationale [12]. For example, the preference for splitting liquefied drug solutions rather than powder arises because it is virtually impossible to split a small amount of powder accurately, while liquefied drug solutions can be divided very accurately using the calibrations on a syringe barrel [12, 24].

Our findings emphasize the need to improve access to sterile injecting equipment. Tajikistan reported distributing 214 syringes per PWID in 2014 [4], thus exceeding targets recommended by the UN technical agencies [25]. Nevertheless, as the participants’ accounts imply, this level of coverage may still be insufficient due to relatively high frequency of injection and replacement of broken and clogged needles and syringes while preparing and injecting heroin. Furthermore, given limited geographical coverage and hours of operation existing NSPs alone are unlikely to meet PWID needs, and therefore, should be complemented by pharmacy-based harm reduction services [26]. Last but not least, since withdrawal was cited as the main reason for using non-sterile equipment, expanding opioid substitution therapy (OST) services and lowering their threshold is another viable strategy to reduce risky injection practices [27]. Combination of OST and NSP, and adequate coverage of these interventions have been identified as critical elements for effective and cost-effective response to both HIV and HCV epidemics among PWID [28, 29].

Finally, other authors suggested that in the absence of a needle and syringe, some users might snort or smoke heroin [30]. However, our results point to injecting as the sole route of administration among drug injectors in Kulob and Khorog.

### Limitations

The sample was primarily NSP clients from two cities and caution should be used in generalizing the findings to PWID who are not NSP clients or to PWID who live in other cities. PWID who do not obtain syringes from NSP may be less knowledgeable regarding safe injecting practices, have less access to sterile injection equipment, and may engage in risky behaviors more frequently. The absence of women in the sample may further reduce the generalizability of the findings. In other locations, significantly more females than males reported sharing needles/ syringes and paraphernalia [11]. In a sample of PWID in Kulob and Khudjand (27 females and 173 males) HIV prevalence and rates of risk behaviors in women were significantly higher than among men (unpublished data). This raises the possibility that our findings may have been different if our sample had included females who inject drugs in addition to males. Despite these limitations, our research adds to the scant literature on injection risk behaviors and associated environmental factors among PWID in Tajikistan. We identified no observable differences in drug injection practices and risk behaviors between two cities, which suggests that the results may be generalizable to the population of NSP clients in other cities in Tajikistan. This information could prove useful for developing more focused harm reduction interventions to reduce injection risk behaviors among PWID in Tajikistan.

## Conclusions

This study highlighted important issues for further research and planning risk reduction activities in Tajikistan. Of particular concern is the limited access to sterile equipment in remote areas and the lack of awareness among PWID regarding risks of indirect sharing (e.g., sharing cookers, cottons, and using a common syringe to divide drug solution). Future harm reduction interventions will need to focus on the different stages of drug preparation and injecting processes, improve access to new needles and syringes and promote safer injecting practices that incorporate current knowledge about the spread of disease through the sharing of drug paraphernalia.

The study also underscores the importance of qualitative research in understanding the social, cultural and economic contexts in which drug use occurs and behavioral norms are shaped. This research helps to identify and interpret risk behaviors associated with injecting drug use and to inform prevention program development and implementation, so that the planned interventions are meaningful and useful to drug users themselves. It is imperative to incorporate qualitative research into design and evaluation of interventions targeting substance-using populations.

Oral informed consent was obtained from the study participants for publication of this report and any accompanying images.
